# Opium Treatment of Rheumatism

**Published:** 1846-06

**Authors:** 


*Opium Treatment of Rheumatism.*—The most important rule to be remembered in employing opium for the cure of acute rheumatism is, that *full and sufficient doses* shall be exhibited. I have heard of the opiate treatment having disappointed those who have tried it. On inquiry, I have learned that in those cases it has been given only to the extent of a grain every fourth or sixth hour. This is not the treatment of rheumatism by opium; it is making the patient worse than before; it is inflicting on the patient the mischief arising from the stimulant effects of the drug, and withholding from him all the benefits of its sedative influence. The opium should always be increased in dose, both as to frequency and quantity, until the patient feels entire relief; and should be then kept up at that dose until the complaint is steadily declining. *Dr. Corrigan* in *Med. Exam.*

